# Repurposing of the Fasciolicide Triclabendazole to Treat Infections Caused by *Staphylococcus* spp. and Vancomycin-Resistant Enterococci

**DOI:** 10.3390/microorganisms9081697

**Published:** 2021-08-10

**Authors:** Hongfei Pi, Abiodun D. Ogunniyi, Bhumi Savaliya, Hang Thi Nguyen, Stephen W. Page, Ernest Lacey, Henrietta Venter, Darren J. Trott

**Affiliations:** 1 Australian Centre for Antimicrobial Resistance Ecology, Roseworthy Campus, School of Animal and Veterinary Sciences, The University of Adelaide, Roseworthy, SA 5371, Australia; hongfei.pi@adelaide.edu.au (H.P.); david.ogunniyi@adelaide.edu.au (A.D.O.); bhumi.savaliya@adelaide.edu.au (B.S.); hang.t.nguyen@adelaide.edu.au (H.T.N.); 2 Department of Pharmacology, Toxicology, Internal Medicine and Diagnostics, Faculty of Veterinary Medicine, Vietnam National University of Agriculture, Hanoi 100000, Vietnam; 3 Luoda Pharma Pty Ltd., Caringbah, NSW 2229, Australia; swp@advet.com.au; 4 Microbial Screening Technologies Pty Ltd., Smithfield, NSW 2164, Australia; elacey@microbialscreening.com; 5 Health and Biomedical Innovation, Clinical and Health Sciences, University of South Australia, Adelaide, SA 5000, Australia; rietie.venter@unisa.edu.au

**Keywords:** multidrug resistance, triclabendazole, polymyxin B, bacterial pathogens, bioluminescence, sepsis

## Abstract

One approach to combat the increasing incidence of multidrug-resistant (MDR) bacterial pathogens involves repurposing existing compounds with known safety and development pathways as new antibacterial classes with potentially novel mechanisms of action. Here, triclabendazole (TCBZ), a drug originally developed to treat *Fasciola hepatica* (liver fluke) in sheep and cattle, and later in humans, was evaluated as an antibacterial alone or in combination with sub-inhibitory concentrations of polymyxin B (PMB) against clinical isolates and reference strains of key Gram-positive and Gram-negative bacteria. We show for the first time that in vitro, TCBZ selectively kills methicillin-sensitive and methicillin-resistant *Staphylococcus aureus* and *Staphylococcus pseudintermedius* at a minimum inhibitory concentration (MIC) range of 2–4 µg/mL, and vancomycin-resistant enterococci at a MIC range of 4–8 µg/mL. TCBZ also inhibited key Gram-negative bacteria in the presence of sub-inhibitory concentrations of PMB, returning MIC_90_ values of 1 µg/mL for *Escherichia coli*, 8 µg/mL for *Klebsiella pneumoniae*, 2 µg/mL for *Acinetobacter baumannii* and 4 µg/mL for *Pseudomonas* *aeruginosa*. Interestingly, TCBZ was found to be bacteriostatic against intracellular *S. aureus* but bactericidal against intracellular *S. pseudintermedius*. Additionally, TCBZ’s favourable pharmacokinetic (PK) and pharmacodynamic (PD) profile was further explored by in vivo safety and efficacy studies using a bioluminescent mouse model of *S. aureus* sepsis. We show that repeated four-hourly oral treatment of mice with 50 mg/kg TCBZ after systemic *S. aureus* challenge resulted in a significant reduction in *S. aureus* populations in the blood to 18 h post-infection (compared to untreated mice) but did not clear the bacterial infection from the bloodstream, consistent with in vivo bacteriostatic activity. These results indicate that additional pharmaceutical development of TCBZ may enhance its PK/PD, allowing it to be an appropriate candidate for the treatment of serious MDR bacterial pathogens.

## 1. Introduction

The increasing public health threat posed by antimicrobial resistance (AMR) and the rise in the incidence of multidrug-resistant (MDR) bacterial pathogens has stimulated novel research strategies to combat MDR infections. However, the discovery, development and marketing approval process for new drugs is very challenging and can cost over 2.8 billion USD and take over 10 years to reach the market [1]. There is a high risk of failure (82.7%) in the preclinical stage alone, which may take up to 6 years [2,3,4].

Repurposing currently registered drugs for new indications is one of the alternative approaches to overcoming these challenges whilst adhering to the principles of antimicrobial stewardship [5,6,7,8]. Using existing drugs with known toxicological and pharmacokinetic profiles and an acceptable level of safety and tolerability is a much cheaper and efficient option than developing entirely new antibiotics [9,10,11] and has become a more common strategy in recent years [4,12,13,14]. One such agent is triclabendazole (TCBZ), a benzimidazole anthelminthic agent, with narrow spectrum activity against trematodes in the genus *Fasciola*, approved for use in animals (especially cattle, sheep and goats) [15,16,17] and humans [18,19,20]. TCBZ has previously been identified as having potential antibacterial activity against Gram-positive bacteria when it was included in a mass screening of Food and Drug Administration (FDA)-approved non-antibacterial drugs [21], and has more recently been shown to have anticlostridial activity [22].

The mechanism by which TCBZ exhibits its effect against *Fasciola* species is not fully elucidated. Both in vitro and in vivo studies suggest that TCBZ and its metabolites (sulfoxide and sulfone) are absorbed across the tegument of immature and mature worms, leading to a decrease in the resting membrane potential, inhibition of tubulin function and inhibition of protein and enzyme synthesis. These metabolic disturbances are associated with inhibition of motility, disruption of cell wall ultrastructure and inhibition of spermatogenesis and vitelline cells [18].

Given indications that TCBZ has potential as an antibacterial agent, to our knowledge, there have been no published studies that directly demonstrate this potential in vivo. A preliminary evaluation of in vitro efficacy of a variety of TCBZ derivatives was therefore carried out against representative Gram-positive (methicillin-resistant and methicillin-susceptible *Staphylococcus* spp., vancomycin-resistant *Enterococcus* spp. and *Streptococcus pneumoniae*) and Gram-negative organisms (*Escherichia coli, Klebsiella pneumoniae, Pseudomonas aeruginosa* and *Acinetobacter baumannii*), including in the presence of polymyxin B (PMB). PMB was used as it is a well-known Gram-negative outer membrane permeabilizer [23,24] and because we have successfully used it for this purpose in previous published work [25]. From the collection of TCBZ and metabolites, TCBZ was chosen as the most active, and further antibacterial activity was explored in vitro. Efficacy testing using a mouse bioluminescent *S. aureus* infection model was also undertaken to better investigate its antibacterial properties in vivo.

## 2. Materials and Methods

### 2.1. Antimicrobial Agents

**Figure 1:**
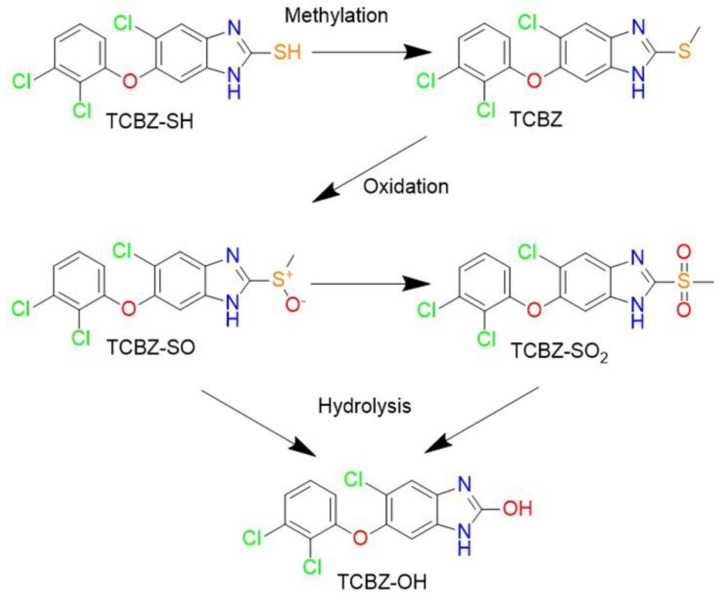
TCBZ and related synthetic precursor, oxidation and hydrolytic analogs.

TCBZ and four derivatives (TCBZ-SH, TCBZ-SO, TCBZ-SO_2_ and TCBZ-OH; Figure 1) were prepared by conjugation of 5-chloro-2-nitroaniline [26] with 2,3-dichlorophenol [27], followed by reduction to the diamine using sodium dithionite and cyclization, which gave TCBZ-SH [28]. TCBZ-SO and TCBZ-SO_2_ were prepared by oxidation with hydrogen peroxide in a mixture of acetic acid and chloroform at a ratio of 4:1, while TCBZ-OH was prepared by reaction of the diamine with potassium cyanate [29]. These analogs were stored in a sealed sample container out of direct light at 4 °C in the Microbiology Laboratory at Clinical and Health Sciences, City East Campus, University of South Australia, Australia.

PMB was prepared as a stock solution of 25.6 mg/mL in DMSO, stored in 1 mL aliquots at −80 °C and defrosted immediately prior to use. TCBZ as Fasinex^®^ 240 Oral Flukicide for Cattle (240 g/L) was purchased from Elanco Australia Pty Ltd (Macquarie Park, NSW, Australia) and stored at room temperature in the original container, tightly closed in a cool dry place. Fresh stock solutions of TCBZ (50 mg/kg and 10 mg/kg) were prepared as oral treatments and diluted in phosphate-buffered saline (PBS) with vigorous mixing. Moxifloxacin hydrochloride was purchased from Bayer Ltd. as Avelox 400 mg in a 250 mL solution (equivalent to 1.6 mg/mL moxifloxacin hydrochloride) and stored at room temperature.

### 2.2. Bacterial Strains

A total of 138 bacterial isolates (55 Gram-positive and 83 Gram-negative) were collected from government, private and university diagnostic laboratories throughout Australia (Table S1). The Gram-positive bacterial collection included 4 vancomycin-resistant enterococci (VRE), 24 *S. aureus* isolates (including 21 methicillin-resistant *S. aureus* (MRSA)), 2 *S. pneumoniae*, 13 *S. pseudintermedius* (including 10 methicillin-resistant *S. pseudintermedius* (MRSP)), 3 coagulase-negative *Staphylococcus* spp. (CoNS) and 9 *Streptococcus* spp. isolates from cases of bovine mastitis. The Gram-negative bacteria collection included 21 *E. coli*, 20 *K. pneumoniae*, 18 *A. baumannii*, 20 *P. aeruginosa*, 2 *Neisseria meningitidis* and 2 *N. gonorrhoeae* isolates. All organisms were identified to species level using biochemical testing and Matrix-Assisted Laser Desorption/Ionization Time-of-Flight (MALDI-TOF) mass spectrometry (Microflex^TM^ LT/SH BioTyper Bruker Daltonics, Leipzig, Germany) at the Australian Centre for Antimicrobial Resistance Ecology (ACARE), The University of Adelaide, Australia.

### 2.3. Minimum Inhibitory Concentration (MIC) Determination

Minimum inhibitory concentrations (MICs) were determined in round-bottom 96-well microtiter trays (Sarstedt 82.1582.001; Mawson Lakes, SA, Australia), using the modified broth microdilution method recommended by the Clinical and Laboratory Standards Institute [30]. Testing concentrations were as follows: TCBZ-256 to 0.25 µg/mL; PMB-32 to 0.06 µg/mL. Cation-adjusted Mueller–Hinton broth (CAMHB; Becton Dickinson, Sparks, MD, USA) was used for MIC testing against all organisms except *S. pneumoniae* and other *Streptococcus* spp., for which CAMHB supplemented with 3% lysed horse blood and 5% horse serum was used. To test the effect of ultra-heat-treated (UHT) milk on the MICs of TCBZ against *Streptococcus* spp., CAMHB supplemented with 3% lysed horse blood and 10% UHT milk was used. To test the effect of pH on the MICs of TCBZ against *Streptococcus* spp., RPMI 1640 medium (Sigma-Aldrich, NSW, Australia) was used instead of CAMHB.

Serial two-fold dilutions of the compounds were performed in 100% DMSO, with 1 µL added to each well. In selected experiments, the MICs for ampicillin, daptomycin, gentamicin and apramycin against each isolate were determined as an internal quality control. The MICs against all isolates were determined by visual reading and using an EnSpire Multimode Plate Reader 2300 (PerkinElmer) at A_600 nm_. MIC_50_, MIC_90_ and MIC ranges for TCBZ, PMB or combinations were then determined [31].

### 2.4. Synergy Testing by Checkerboard Microdilution and Dose Reduction Analysis

The potential activity of TCBZ against clinical and ATCC Gram-negative pathogens was determined in the presence or absence of 0.25–128 µg/mL PMB in a modified standard checkerboard assay as described previously [32,33]. Briefly, antimicrobial stock solutions were prepared at a concentration of 12.8 mg/mL in DMSO for TCBZ and 12.8 mg/mL in Milli-Q water for PMB. Then, a two-fold serial dilution of each antimicrobial stock solution was prepared in its appropriate solvent from wells 12 to 3 (from 12.8 to 0.25 mg/mL) and 1 µL of each concentration was added to each well in the challenge plate using an electronic multichannel pipette followed by 89 µL of the LB broth. Thereafter, 10 µL of bacterial suspension of approx. 2 × 10^6^ colony-forming units per milliliter (CFU/mL) was added to each well of the plate, which was subsequently incubated at 37 °C for 24 h.

The fractional inhibitory concentration index (FICI) described the results of combination and was calculated as follows: FICI of combination = FIC A + FIC B. Where FIC A is the MIC of TCBZ in the combination/MIC of TCBZ alone, FIC B is the MIC of PMB in the combination/MIC of PMB alone [25,33]. The results indicate synergism when the corresponding FICI ≤ 0.5, additivity when 0.5 < FICI ≤ 1, indifference when 1 < FICI ≤ 4 and antagonism when the FICI > 4. In this study, the FICI for TCBZ and PMB against Gram-negative bacteria was calculated to be zero (e.g., 1 ÷ > 256 = 0) where they did not show any antibacterial activity alone against Gram-negative bacteria at the highest concentration (256 µg/mL).

The dose-reduction index (DRI) shows the difference between the effective doses in combination in comparison to its individual dose. DRI was calculated as follows: DRI = MIC of drug alone/MIC of drug in combination. Given that TCBZ did not show any antimicrobial activity against the majority of Gram-negative bacteria, the highest concentration of each compound tested against each isolate was used as its MIC alone for calculating the DRI (e.g., MIC of TCBZ alone against *E. coli* 10763 was > 256 μg/mL and its MIC in combination with PMB was 1 μg/mL; DRI = 256/1). DRI is significant clinically when the dose reduction is associated with a toxicity reduction without changing efficacy [34]. Commonly, a DRI higher than 1 is considered beneficial.

### 2.5. Minimum Bactericidal Concentration (MBC) Determination

The minimum bactericidal concentration (MBC) for TCBZ against Gram-positive bacteria alone or against Gram-negative bacteria (in combination with PMB) were determined as follows. Briefly, 10 µL aliquots from each duplicate well from the MIC assays (starting from the MIC for each compound) were inoculated onto a sheep blood agar (SBA) plate and incubated at 37 °C. Plates were examined at 24 and 48 h and the MBC was recorded as the lowest concentration of each test compound at which a 99.9% colony count reduction was observed on the plate [34].

### 2.6. Time-Dependent Killing Assays

Time-kill assays were performed (in duplicate) for TCBZ against Gram-positive bacteria (MRSP-1, *S. aureus* ATCC 29213 and VRE 35C) and for TCBZ in the presence of PMB against Gram-negative bacteria (*E. coli* ATCC 25922) as described previously [34]. Briefly, a few colonies of each strain from overnight SBA plates were emulsified in normal saline and adjusted to *A*_600 nm_ = 0.10 (equivalent to approx. 5 × 10^7^ CFU per mL) and the bacterial suspensions were further diluted 1:20 in saline. TCBZ and PMB were serially diluted in 100% DMSO or Milli-Q water at 100 × the final desired concentration and a 100 µL aliquot of each appropriate concentration was added to each 10 mL preparation. TCBZ and PMB solutions were prepared in 10 mL volumes at 1 × MIC, 2 × MIC and 4 × MIC concentrations in LB broth. After adding inoculum dose to each tube, duplicate cultures were incubated at 37 °C, with samples withdrawn at 0, 0.5, 1, 2, 4, 6, 8 and 24 h, serially diluted 10-fold and plated on SBA overnight at 37 °C for bacterial enumeration. According to CLSI, an antimicrobial agent is considered bactericidal if it causes a ≥ 3 × log_10_ (99.9%) reduction in colony-forming units per milliliter (CFU/mL) after 18–24 h of incubation, and the combination is considered synergistic when it causes a ≥ 2 × log_10_ reduction in CFU/mL.

### 2.7. Multi-Sub-Culture Resistance Selection

Multi-sub-culture resistance selection studies (21 days sequential culturing of *S. aureus* ATCC 29213) were undertaken using enrofloxacin as a control antibiotic, as described previously [35]. Each day an MIC test concentration range of antibiotics from 0.25 to 8 µg/mL on 48 well plates was used. On the first day, the same method described for MIC microdilution was used to determine the MIC and the plate was incubated at 37 °C for 24 h. Sub-cultures were performed at 24 h intervals for up to 21 days by transferring 20 µL aliquot of culture, containing 5 × 10^5^ CFU, from the well nearest the MIC (usually 1 to 2 dilutions below) which had the same turbidity as antibiotic-free controls. During the sub-culture, the resistant mutants that emerged were stored at −80 °C for subsequent analysis.

### 2.8. Intracellular MIC Testing

The mouse J774A.1 macrophage cell line was maintained in DMEM (Dulbecco’s Modified Eagle Medium) with heat-inactivated fetal bovine serum in cell culture flasks (75 cm^2^), as described previously [36]. Cells were allowed to grow to 90% confluency and were then passaged and transferred to a 48-well cell culture plate. Cells were then allowed to grow up to a concentration of 5–8 × 10^5^ cells/mL.

*S. aureus* strain ATCC 29213 was sub-cultured on SBA and incubated at 37 °C for 18–20 h. A number of isolated colonies were then resuspended in RPMI medium, adjusted to 0.5 McFarland standard and opsonized in 10% freshly collected human serum (stored at −-80 °C until use) at 37 °C for 30 min. Cell culture medium was replaced with the opsonized bacterial suspension to allow phagocytosis at 37 °C for 1 h at a macrophage:bacterium ratio of 1:2. Macrophage cells were then washed twice in DMEM to remove extracellular bacteria and exposed to TCBZ at concentrations ranging from 0.5–32 µg/mL, while cells were also exposed to monensin at concentrations ranging from 0.25–16 µg/mL as a control. At this stage, 50 µg/mL gentamicin was added to kill any remaining extracellular bacteria and the infected macrophage cells were incubated for a further 20–24 h. After incubation, infected macrophage cells were washed with PBS three times to remove any carryover antibiotics and then collected by scraping. Two protocols were then undertaken in parallel: (i) after 1:10, 1:100 and 1:1000 dilutions, 10 µL aliquots of the harvested cells were spot-plated onto SBA for determination of CFU. Plates were then incubated at 37 °C for 24 h. The colonies were counted the next day and CFUs were determined. (ii) The protein concentration was determined as per manufacturer’s instructions with bovine serum albumin as a standard (DC protein assay kit, Bio-Rad Catalog No 500-0112). All results were then calculated as CFU/mg of cell protein and these values were plotted using ‘R’ software (version: 3.6.1).

### 2.9. Haemolysis Assay

This was performed using fresh human red blood cells (RBCs) from donors as described previously [25,37]. Fresh RBCs were washed in PBS three times at 500 × g for 5 min, and then resuspended 1% (*w*/*v*) in PBS. Serial two-fold dilutions of each compound (2 µL each) were added into the respective wells, in quadruplicates, in a round-bottom 96-well microtiter tray (Sarstedt 82.1582.001; Mawson Lakes, SA, Australia), starting at 128 µg/mL for each compound using ampicillin as a control. Thereafter, 198 µL of the 1% RBC solution was added to each well, and the mixture was incubated for 1 h at 37 °C with shaking at 100 rpm. Quadruplicate wells containing either 1% Triton X100 or PBS only served as controls. After incubation, the trays were centrifuged at 1000 × g for 3 min and 100 µL of supernatant from each well was transferred into a new 96-well tray. Absorbance was measured at *A*_450nm_ on a Cytation 5 Cell Imaging Multi-Mode Reader (BioTek, Millennium Science Pty Ltd., Mulgrave, VIC, Australia) and plotted against each dilution. Hemolytic titer was determined as the reciprocal of the dilution at which 50% of erythrocytes were lysed at *A*_450nm_.

### 2.10. In Vitro Cytotoxicity Assays

We assayed TCBZ for in vitro cytotoxicity using a panel of adherent mammalian cell lines HEK293 (human embryonic kidney cell line), Detroit 562 (human nasopharyngeal carcinoma epithelial cell line) and MCF-7 (human mammary gland adenocarcinoma cell line), as described previously [35]. Assays were performed in duplicates in flat-bottom black 96-well tissue culture trays (Costar) seeded with ~1 × 10^4^ cells per well. After 24 h incubation, media was removed, washed once with medium without antibiotics and fresh medium supplemented with 10% (vol/vol) FBS was added. Viability of each cell line in the presence of each compound was assessed starting at 64 μg/mL (for HEK293 and Detroit 562 cell lines) or starting at 16 μg/mL (for MCF-7) at 1 h intervals for 20 h at 37 °C and in 5% CO_2_ on a Cytation 5 Cell Imaging Multi-Mode Reader (BioTek, Millennium Science Pty Ltd., Mulgrave, VIC, Australia) using the RealTime-Glo™ MT Cell Viability Assay reagent (Promega, Madison, WI, USA).

### 2.11. Ethics Statements

For TCBZ safety and efficacy testing experiments, outbred 5- to 6-week-old male CD1 (Swiss) mice (weighing between 25 g to 32 g) obtained from the Laboratory Animal Services breeding facility of the University of Adelaide were used. Mice had access to food and water ad libitum throughout the experiments. The Animal Ethics Committee of The University of Adelaide (approval number S-2015-151) reviewed and approved all animal experiments. The studies were conducted in compliance with the Australian Code of Practice for the Care and Use of Animals for Scientific Purposes (8th Edition 2013) and the South Australian Animal Welfare Act 1985.

### 2.12. Oral Safety Assessment of TCBZ following Parenteral Administration

Mice were placed in individually ventilated cages in 3 treatment groups as follows: (i) 10 mg/kg of TCBZ (*n* = 3); (ii) 50 mg/kg of TCBZ (*n* = 3); (iii) PBS (*n* = 3); and (iv) 1.6 mg/mL of moxifloxacin (*n* = 3). Mice in group (i) were treated with a total of 25 µL of 10 mg/kg of TCBZ. Mice in group (ii) were treated with 125 µL of 50 mg/kg of TCBZ, mice in group (iii) were treated with 25 µL of PBS while mice in group (iv) were treated with a total of 94 µL of moxifloxacin. All mice were treated three times per day for 5 days. At the conclusion of the experiment (5 days from the start), mice were humanely killed and sections of tissues (lung, heart, liver, spleen, kidney and small intestinal) were collected and subjected to histopathological analysis.

### 2.13. Histopathological Examination

Mouse tissues (including lung, heart, liver, spleen, kidney and small intestinal) collected from the oral safety challenge were fixed in 10% neutral-buffered formalin and processed routinely. The specimens were embedded in paraffin blocks and sections of 4 µm thickness were cut using a microtome. Hematoxylin and Eosin staining of the sections were performed and the slides were observed and recorded under light microscopy.

### 2.14. Oral Efficacy Testing of TCBZ following Systemic Challenge of Mice with Bioluminescent Gram-Positive Bacteria

For oral efficacy testing experiments against *S. aureus*, bioluminescent ATCC12600 strain (Xen29, PerkinElmer) was used as described previously [25,38], but with some modifications. Bacteria were grown in LB broth at 37 °C to A_600 nm_ of 0.5 (equivalent to approx. 1.5 × 10^8^ CFU/mL). Three groups of mice (*n* = 6 mice per group) were challenged IP with approx. 2.5 × 10^7^ CFU (first experiment) or approx. 1 × 10^7^ CFU (second experiment) of Xen29 in 200 µL PBS containing 3% hog gastric mucin type III (Sigma Aldrich, NSW, Australia). At 2 h post-infection, approx. 50 µL of blood was withdrawn from the submandibular plexus of all mice for bacterial enumeration after which they were subjected to bioluminescence imaging in both ventral and dorsal positions on the IVIS Lumina XRMS Series III system (Caliper LifeSciences, Hopkinton, MA, USA). Immediately thereafter, group 1 mice received 25 µL of PBS orally, group 2 mice received 125 µL of TCBZ at 50 mg/kg orally and group 3 mice received 94 µL of moxifloxacin orally. At 6 h, 10 h, 18 h and at 72 h post-infection (or during humane killing of moribund mice), approx. 50 µL blood was again withdrawn, followed by bioluminescence imaging. After imaging at 6 h post-infection, a second dose of PBS/TCBZ/moxifloxacin was administered. Further treatments were given at 10 h, 18 h and 24 h post-infection. Mice were monitored frequently (every 4 h) for signs of distress throughout the experiment, and the clinical conditions were recorded on a clinical record sheet approved by The University of Adelaide Ethics Committee. Mice that had become moribund or showed any evidence of distress were humanely euthanized by cervical dislocation. 

The first experiment showed a lack of efficacy for TCBZ post-24 h, which was partly due to the high bacterial challenge dose. Therefore, a repeat experiment using a lower bacterial dose (1 × 10^7^ of Xen29) (*n* = 6 mice per group) was carried out as described above. In both experiments, signals were collected from a defined region of interest and total flux intensities (photons/s) were analyzed using Living Image Software 4.5. Differences in luminescence signals between control and drug-treated groups were compared by the Mann–Whitney U test (one-tailed).

## 3. Results 

### 3.1. TCBZ Derivatives Demonstrate Antibacterial Activity

The potential of TCBZ as an antibacterial agent was initially examined by conducting MIC testing of five of its derivatives against two VRE isolates and two *S. aureus* isolates. The MIC determination showed that three of the five analogs possessed antimicrobial activity against the four Gram-positive isolates selected (Table 1).

MICs were repeated with the parent compound (TCBZ) and its thiol (TCBZ-SH) and sulphoxide (TCBZ-SO) derivatives showing promising activity against three VRE, three MRSA and two *S. pneumoniae* isolates, essentially confirming the initial results (Table 2). Notably, only TCBZ showed antimicrobial activity against both *S. pneumoniae* isolates. Therefore, TCBZ was chosen as the most promising analog for further evaluation.

### 3.2. TCBZ Shows Antimicrobial Activity against an Expanded Range of Staphylococcus *spp.* and Vancomycin-Resistant Enterococci

Given the promising antibacterial activity of TCBZ in the initial screen, its spectrum of activity was further investigated against a range of clinical coagulase-positive *Staphylococcus* spp., including human MRSA (*n* = 20; Table 3 and Table S2) and canine MRSP (*n* = 13; Table S3) isolates. TCBZ inhibited the growth of all tested *S. aureus* and *S. pseudintermedius* including MRSA and MRSP isolates at concentrations ranging from 2–4 µg/mL. The MBC values of TCBZ against the *S. aureus* and *S. pseudintermedius* isolates were between 1–4 times the MIC values, confirming that TCBZ is bactericidal against coagulase-positive *Staphylococcus* spp. (Table S3). However, in the presence of UHT milk, TCBZ did not show any antimicrobial activity against *S. aureus* ATCC29213 at the highest concentration (64 µg/mL) tested.

### 3.3. TCBZ Shows Limited Antimicrobial Activity against Streptococcus *spp.* Causing Bovine Mastitis

We initially obtained a MIC of 16 µg/mL activity for TCBZ against two *S. pneumoniae* clinical isolates. Therefore, further evaluation of its activity against other streptococci causing bovine mastitis (*S. uberis*, *S. agalactiae* and *S. dysgalactiae*) was conducted. However, no antimicrobial activity was detected for these streptococci, except for a single isolate of *S. dysgalactiae* (MIC and MBC both 32 µg/mL). In addition, the effect of ultra-heat-treated (UHT) milk in CAMHB medium and pH (RPMI medium) on the MIC of TCBZ against the *S. uberis, S. agalactiae* and *S. dysgalactiae* isolates was also determined; however, no antimicrobial activity was detected.

### 3.4. TCBZ in Combination with PMB Demonstrates Synergistic Activity against a Range of Gram-Negative ESKAPE Pathogens

The antimicrobial activity of TCBZ in the presence of PMB was investigated against a range of clinical and reference human ESKAPE pathogens. These included *E. coli* strains ATCC 10763 and ATCC 25922, *P. aeruginosa* strain PAO1, *K. pneumoniae* strains ATCC 33495 and ATCC 4352, *A. baumannii* strains ATCC 19606 and ATCC 12457, *N. meningitidis* clinical isolates 423 and 424 as well as *N. gonorrhoeae* strains ATCC 16599 and ATCC 49226. The combination of TCBZ and PMB resulted in a synergistic interaction against all the isolates tested except for *N. meningitidis* and *N. gonorrhoeae* (Table 4).

The antimicrobial activity of the TCBZ and PMB combination was tested against a larger collection of human ESKAPE pathogens (18 *K. pneumoniae* clinical isolates plus ATCC 33495 and ATCC 4352, 18 *E. coli clinical* isolates plus ATCC 10763 and ATCC 25922, 16 *A. baumannii* clinical isolates plus *A. baumannii* ATCC 19606 and NCIMB 12457 as well as 19 *P. aeruginosa* clinical isolates plus PAO1) (Table 5). The results reveal a synergistic or additive interaction of TCBZ and PMB against all Gram-negative isolates tested (reducing the MIC of TCBZ by 16- to 2048-fold against all Gram-negative species tested).

### 3.5. TCBZ Kinetic Assays Confirm Bactericidal Activity 

**Figure 2:**
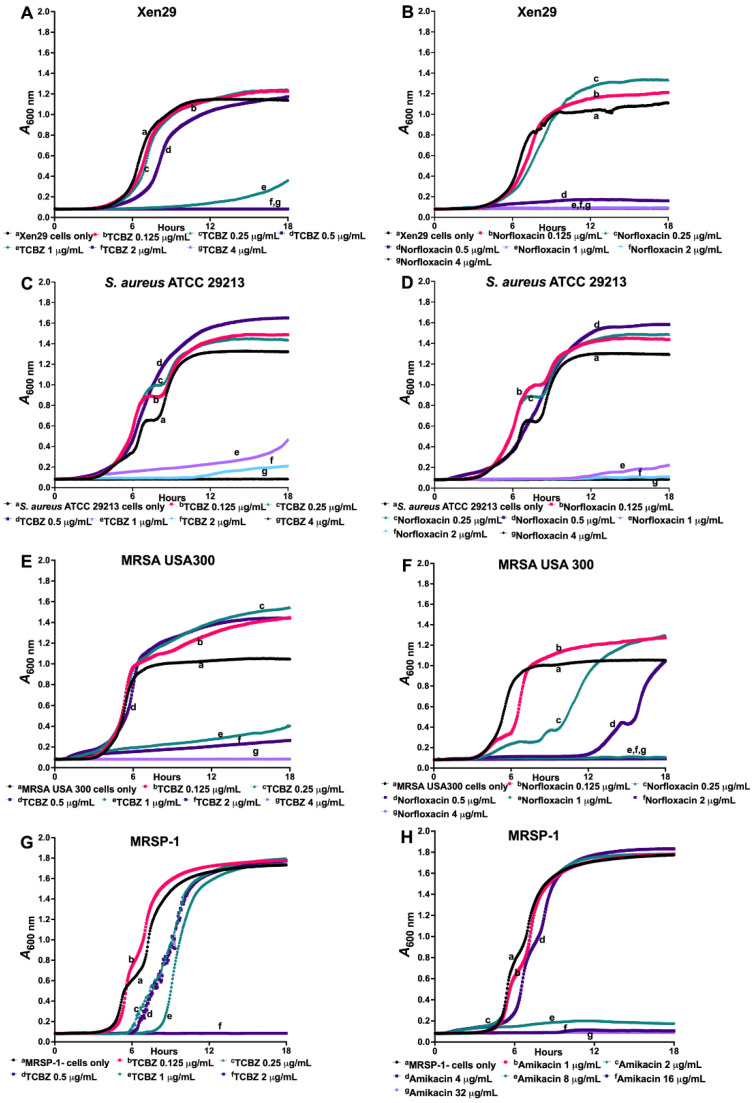
Kinetic assay showing time- and concentration-dependent inhibition of *S. aureus* Xen29 (**A**,**B**), *S. aureus* ATCC 29213 (**C**,**D**), MRSA USA300 (**E**,**F**) and MRSP-1 (**G**,**H**), using norfloxacin or amikacin as a comparator. The results show TCBZ is bactericidal against the four bacteria tested.

TCBZ was further investigated in kinetic assays to measure the time- and concentration-dependent activity against *S. aureus* Xen 29 (Figure 2A,B), *S. aureus* ATCC 29213 (Figure 2C,D), MRSA USA300 (Figure 2E,F), and MRSP-1 (Figure 2G,H), using norfloxacin or amikacin as a comparator. The results confirm the MBC results showing that TCBZ is bactericidal against *Staphylococcus* spp.

**Figure 3:**
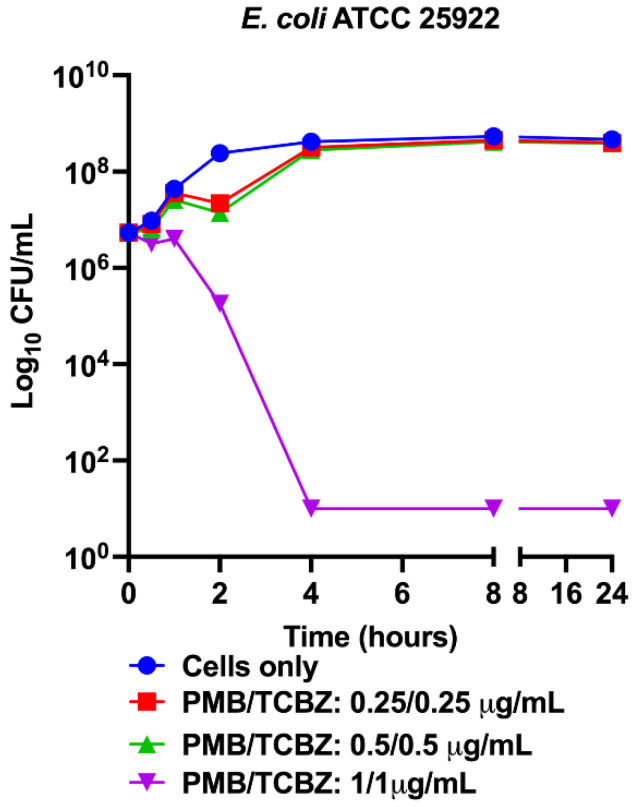
Time-kill curves of TCBZ in combination with PMB against *E. coli* ATCC 25922.

The synergistic activity of TCBZ in combination with PMB against Gram-negative bacteria was further evaluated in a time-kill assay using *E. coli* ATCC 25922. PMB/TCBZ at 0.25/0.25 µg/mL and at 0.5/0.5 µg/mL showed no antimicrobial activity while PMB/TCBZ at 1/1 µg/mL reduced the CFU/mL by 6 × log_10_ by 4 h post-treatment and totally cleared the bacteria, which demonstrated that TCBZ was bactericidal in the combination against *E. coli* ATCC 25922 (Figure 3).

### 3.6. No TCBZ-Resistant Mutants Developed after 21 Daily Sequential In Vitro Sub-Cultures

**Figure 4:**
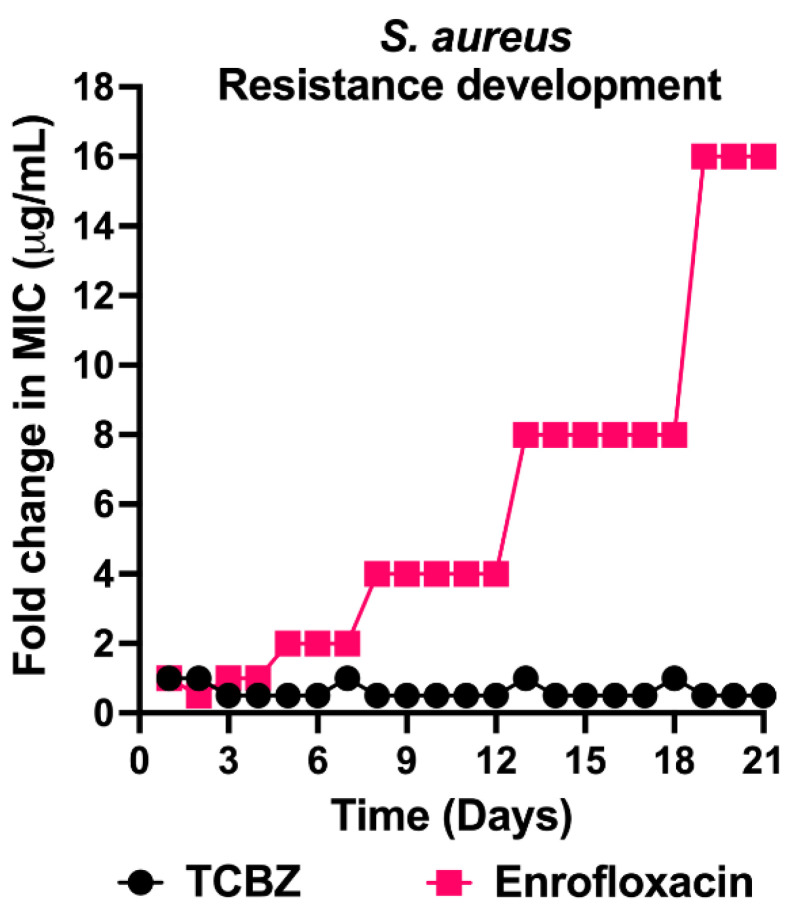
Resistance development of *S. aureus* ATCC 29213 to TCBZ. *S. aureus* ATCC 29213 was challenged with TCBZ at a concentration range of 0.25 to 8 µg/mL over 21 daily sequential in vitro sub-cultures. Enrofloxacin was used as a control antibiotic.

Multi-sub-culture resistance selection was conducted to determine if TCBZ-resistant mutants could develop. For this assay, 21 daily sequential in vitro sub-cultures were performed with TCBZ against *S. aureus* ATCC 29213, using enrofloxacin as a control antibiotic. After 21 days, no TCBZ-resistant mutants were identified. However, enrofloxacin-resistant mutants (2 × the MIC) had developed by day five and increased gradually to 16 × MIC by days 18–21 (Figure 4).

### 3.7. Intracellular MIC Testing of TCBZ Shows Bacteriostatic Activity against S. aureus but Bactericidal Activity against MRSP

**Figure 5:**
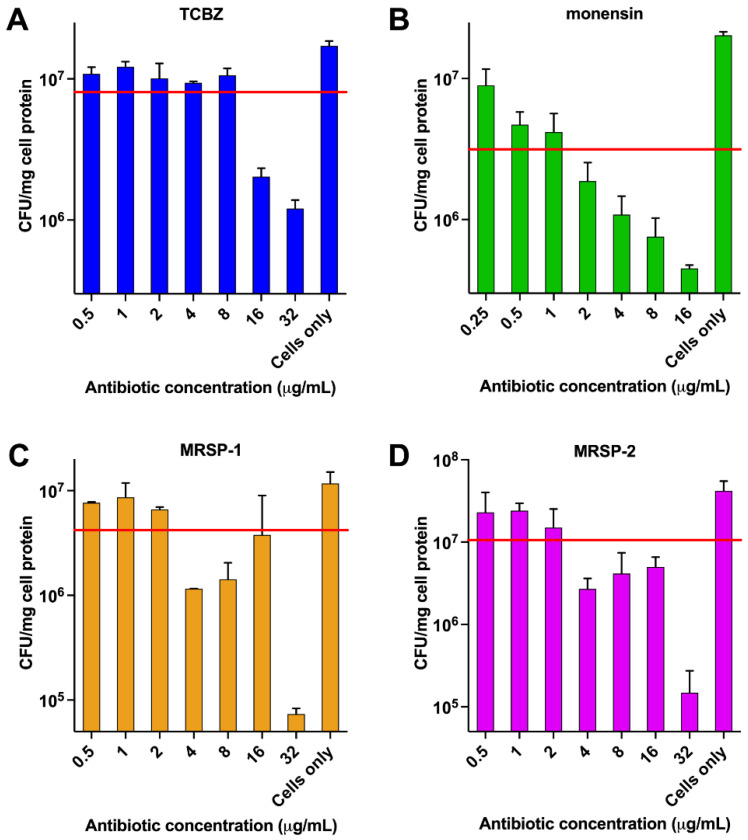
Intracellular activity of TCBZ using mouse J774A.1 macrophage cell line. (**A**) Intracellular activity of TCBZ against *S. aureus* ATCC 29213; (**B**) intracellular activity of monensin against *S. aureus* ATCC 29213; (**C**) intracellular activity of TCBZ against MRSP 1; and (**D**) intracellular activity of TCBZ against MRSP 2.

The intracellular activity of TCBZ against *S. aureus* ATCC 29213 and two MRSP isolates was examined in the mouse J774A.1 macrophage cell line. TCBZ was found to be bacteriostatic against intracellular *S. aureus* ATCC 29213 (Figure 5A). TCBZ exerted the lowest effect on intracellular bacteria with a maximal decrease in bacterial counts of approximately 0.2 log_10_ at a concentration of 8 × MIC. By contrast, the positive control antimicrobial (monensin) revealed a slowly developing bactericidal effect towards intracellular *S. aureus* ATCC 29213 that was concentration dependent. Monensin showed a decrease in bacterial counts of approximately 1.1 log_10_ at 8 × MIC in broth. Monensin showed a 0.5 log_10_ decrease in intracellular bacterial counts when examined at 1 × MIC (2 µg/mL) in broth (Figure 5B).

The intracellular activity of TCBZ against two MRSP isolates (isolate 1 and 2) was also examined. At the highest concentration used (32 µg/mL (16 × MIC)), TCBZ showed a bactericidal effect with a decrease in bacterial counts of 1.1 log_10_ and 1.4 log_10_ for isolate 1 and isolate 2, respectively (Figure 5C,D). Intracellular MRSP isolates were unaffected by TCBZ when cells were incubated with a drug concentration equal to 1 × MIC (2 µg/mL).

### 3.8. TCBZ Is Non-Hemolytic and Non-Cytotoxic

A hemolytic activity assay for TCBZ against fresh human RBCs from donors showed that it is non-hemolytic, with a HC_50_ higher than 128 μg/mL. Cytotoxic assay results also show that the IC_50_ value for TCBZ was greater than the highest concentration tested (16 μg/mL) against the MCF-7 (human breast cancer) cell line, suggesting non-cytotoxicity against this cell line, while the IC_50_ values were 16 μg/mL against the HEK293 (human embryonic kidney) and Detroit 562 (human pharyngeal epithelial) cell lines, suggesting limited cytotoxicity against these two cell lines.

### 3.9. TCBZ Shows Oral Safety in Mice

Safety studies performed on mice using three doses of either 10 mg/kg or 50 mg/kg TCBZ orally daily for 5 days showed no significant pathological changes in the lungs, heart, liver, spleen, kidneys or small intestine of the treated groups compared to the group treated with a similar dosing regimen of 1.6 mg/mL of moxifloxacin or the control group treated with PBS.

### 3.10. Treatment of Mice with TCBZ Reduces S. aureus Population with Repeated Treatments within 24 h Post-Infection

**Figure 6:**
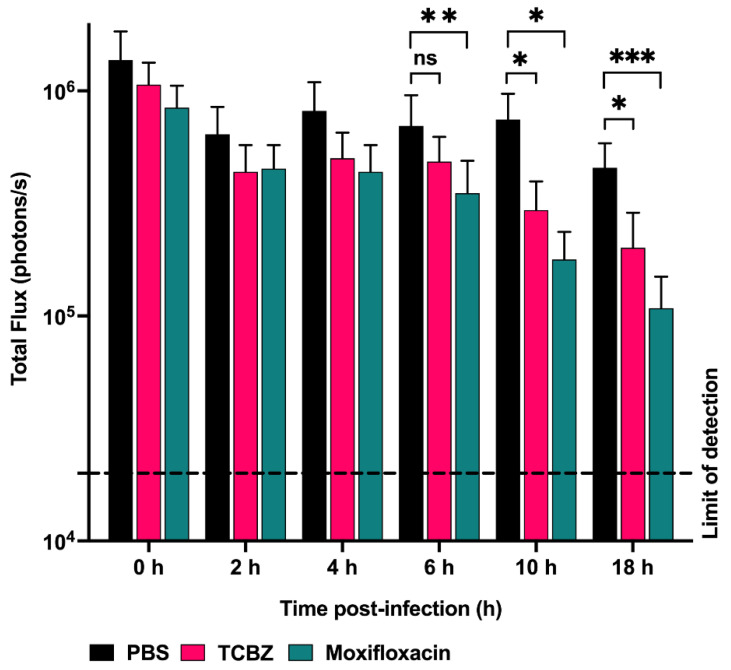
Luminescence signal comparison between groups of CD1 mice challenged IP with 2.5 × 10^7^ CFU of bioluminescent *S. aureus* Xen29 (*n* = 6) (treated at 2, 6, 10 and 18 h post-infection). Mice were subjected to bioluminescence imaging on IVIS Lumina XRMS Series III system. Dashed horizontal line indicates limit of detection (2.0 × 10^4^ photons/s). Data are mean (±SEM) photons/s. * *p* < 0.05; ** *p* < 0.01; *** *p* < 0.001; ns = not significant; Mann–Whitney U test, one-tailed.

**Figure 7:**
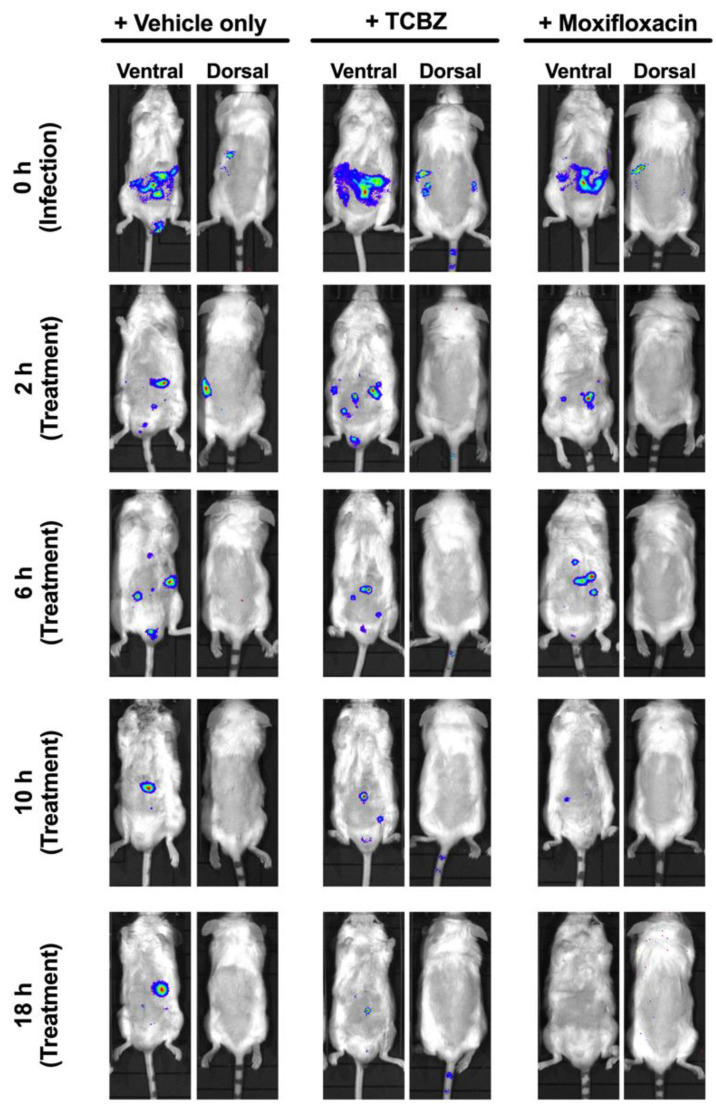
Ventral and dorsal images of representative CD1 mice challenged with 2.5 × 10^7^ CFU of bioluminescent *S. aureus* Xen29. Mice were subjected to bioluminescent imaging on IVIS Lumina XRMS Series III system at the indicated times (0, 2, 6, 10 and 18 h).

We evaluated the potential of TCBZ as an oral drug against systemic *S. aureus* infection, using a well-characterized luminescent strain (Xen29) [25,38], by photon intensity measurements. In the first experiment, mice were challenged IP with 2.5 × 10^7^ CFU of Xen29 and treated with TCBZ at 50 mg/kg or with moxifloxacin at 6 mg/kg orally (administered at 2, 6, 10 and 18 h post-infection), with untreated mice receiving an identical course of PBS orally. The TCBZ treatment regimen resulted in a statistically significant reduction in *S. aureus* populations at 10 and 18 h post-infection (*p* = 0.0483 and *p* = 0.0296, respectively; Mann–Whitney U test, one-tailed) compared to the PBS control group. However, the *S. aureus* populations gradually increased from 24 h post-infection upon withdrawal of TCBZ treatment, consistent with a bacteriostatic action (not shown). Furthermore, mice were found to have severe breathing difficulties and were reluctant to move from 36 h post-infection onwards. Therefore, the mice were humanely killed, blood samples were collected, and no bioluminescent total flux values were recorded for these time points. By contrast, oral administration of moxifloxacin (drug control) resulted in a statistically significant reduction in *S. aureus* populations at 6, 10 and 18 h post-infection (*p* = 0.0042; *p* = 0.0125 and *p* = 0.0006, respectively; Mann–Whitney U test, one-tailed) and there was no regrowth of the bacterial population post-moxifloxacin withdrawal (Figure 6), illustrated by images of representative mice at selected time points (Figure 7).

We attributed the overall lack of efficacy for TCBZ post-24 h to be partly due to the high bacterial challenge dose. Therefore, a repeat experiment using a lower bacterial dose (1 × 10^7^ of Xen29) was carried out. In this experiment, repeated treatment with TCBZ at 50 mg/kg up to 24 h post-infection (administered at 2, 6, 10, 18, and 24 h post-infection) also resulted in a gradual reduction in *S. aureus* populations. However, this reduction was not statistically significant compared to the untreated (PBS) control group (Figure S1). By contrast, oral administration of moxifloxacin (drug control) resulted in a statistically significant reduction in *S. aureus* populations at 18 h post-infection (*p* = 0.0014 and *p* = 0.0031, respectively, Mann–Whitney U test) and there was no regrowth of the bacterial population post-moxifloxacin withdrawal (Figure S1).

## 4. Discussion

The global rise in multidrug-resistant ESKAPE infections continues to pose significant health and economic problems worldwide. However, no novel antibiotic with a new chemical structure, unexploited target or a new mode of action has been developed and marketed for several decades [39,40]. Modification of existing drug classes has resulted in the registration of several new drugs that are active against Gram-positive and some Gram-negative pathogens; however, antimicrobial access through the outer membrane barrier of Gram-negative bacteria still remains an important challenge [37,41,42]. Drugs in advanced clinical development include modification of existing antibiotic classes, new antibiotic classes, well-known combinations or novel drugs with adjuvants. Other recent and promising approaches include repurposing old compounds with known safety and development pathways as new antimicrobial classes with potentially novel mechanisms of action [21], an approach taken here. In developing new broad-spectrum antimicrobial agents that can overcome the permeability barrier in Gram-negative bacterial pathogens [31,43,44], one strategy being employed is combination of antibiotics with outer membrane permeabilizers [45].

In this work, we evaluated the antibacterial potential of TCBZ, a benzimidazole anthelmintic agent, which is the active ingredient of registered drugs for treating liver fluke in animals (*Fasciola* spp.) and lung fluke in humans (*Paragonimus* spp.). TCBZ was previously suggested as a potential antibacterial agent in a repurposing study involving the mass screening of FDA-registered drugs [21]; however, it was not until quite recently that it was demonstrated to have potent antimicrobial activity against *C. difficile* as well as being active against representative strains of normal human gut microbiota [22]. In our study, a range of TCBZ derivatives were generated for an initial screen to identify the candidate with the best antibacterial activity. An investigation of the antibacterial activity of a family of TCBZ analogs incorporating the synthetic precursor thiol, TCBZ’s sequential oxidation sulphoxide (TCBZ-SO) and sulphone (TCBZ-SO_2_), together with the final hydrolytic product (TCBZ-OH), highlighted that the bioactivity is not restricted to the parent anthelmintic but extends to its recognised in vivo metabolites. From the initial screening of the compound series, TCBZ was found to be the most promising derivative with antimicrobial activity demonstrated against Gram-positive bacteria including methicillin-resistant strains, as well as Gram-negative bacteria in the presence of an outer membrane permeabilizer. This corroborates the previous report [22], but also expands the antibacterial spectrum of activity of TCBZ.

The mechanism of action of TCBZ has not been fully clarified. However, in vitro and/or animal infection studies using TCBZ and its active metabolites (sulphoxide and sulfone) against *Fasciola* species point to its effect on multiple targets resulting in reduced membrane potential, inhibition of tubulin function and protein and enzyme synthesis [43]. Interesingly, it appears the sulphoxide metabolite (which is largely predominant in human plasma following pre-systemic biotransformation of TCBZ) has a delayed but more potent effect on parasite motility than the parent TCBZ compound, leading to the suggestion that TCBZ likely acts primarily through the sulphoxide metabolite [46]. It is likely that TCBZ and its metabolites have a similar mechanism of action on the membrane of bacteria, and this will be the subject of future investigations.

This study had four major findings. Firstly, TCBZ was bactericidal against a range of methicillin-susceptible and methicillin-resistant *Staphylococcus* spp. and VRE, and was also shown to maintain antimicrobial activity against intracellular *Staphylococcus* spp. in vitro. Secondly, no resistance to TCBZ was developed by *S. aureus* after 21 daily sequential in vitro sub-cultures, a desirable characteristic for its further exploration as a novel antimicrobial class to treat acute bacterial infections. Thirdly, TCBZ inhibited the growth of key Gram-negative ESKAPE pathogens including *E. coli*, *K. pneumoniae*, *A. baumannii* and *P. aeruginosa* in the presence of sub-inhibitory concentrations of PMB, indicating that the antimicrobial target is present in both Gram-positive and Gram-negative bacteria. Fourthly, repeated oral treatment of mice infected with bioluminescent *S. aureus* with TCBZ significantly reduced *S. aureus* populations within 24 h post-infection, but did not eliminate infection with the selected dosage regimen.

TCBZ demonstrated bactericidal antimicrobial activity (MICs of 2 µg/mL and MBCs ranging from 2–16 µg/mL) against a range of methicillin-susceptible and methicillin-resistant strains of coagulase-positive *Staphylococcus* spp., including the canine commensal pathogen *Staphylococcus pseudintermedius*. Here, MICs/MBCs established for TCBZ were several-fold lower than previously reported for triclabendazole [21], but the same trends were observed (low uniform MICs with MBCs several-fold higher). Furthermore, TCBZ was active at higher concentrations against VRE and *S. pneumoniae*. Demonstrated intracellular activity against *S. aureus* suggested that TCBZ may have potential as a treatment for bovine mastitis, which is most commonly caused by *S. aureus* strains adapted for intracellular survival within polymorphonuclear cells of the bovine mammary gland. However, the promising activity shown against human streptococci did not extend to the three main species of streptococci causing bovine mastitis (*S. uberis*, *S. agalactiae* and *S. dysgalactiae*) and furthermore, antibacterial activity was not demonstrated in the presence of milk, a key disqualifier for further development as an intramammary mastitis formulation. 

Despite these potential drawbacks, an important finding of this work was the demonstration that *S. aureus* did not develop resistance following 21 serial sub-cultures in the presence of sub-inhibitory concentrations of TCBZ. By comparison, serial stepwise resistance was observed for enrofloxacin, a registered fluoroquinolone for animal treatment with broad-spectrum bactericidal antimicrobial activity. Stepwise resistance to fluoroquinolones develops via the accumulation of point mutations in the chromosomal target genes [47]. In this context, our results may indicate that similar chromosomal point mutations in the putative target gene/s of triclabendazole in bacteria may not result in resistance. However, this needs to be further investigated as it is quite possible that point mutations conferring resistance could arise under the right selection pressure. Whilst the exact mechanism of action of triclabendazole in flukes remains to be determined, development of resistance in *Fasciola hepatica* has been recently observed [43]. Further research such as use of differential, timed exposure RNAseq, fluorescence-based protein and lipid binding experiments and electron microscopy studies could potentially unlock the target/s and mechanism of action of triclabendazole in susceptible bacteria.

The observed in vitro synergistic antimicrobial activity of TCBZ in combination with sub-inhibitory concentrations of PMB against a large panel of Gram-negative human ESKAPE pathogens provides additional opportunities for treatment of Gram-negative infections in clinical settings whilst also reducing the amount of PMB needed for effective targeting. Although it was previously reported that PMB is toxic for humans at a concentration of 4 µg/mL, the lowest concentration required in combination with TCBZ ranged from 0.0625 to 0.25 µg/mL, which is 64- to 16-fold lower than its cytotoxic dose [45,48]. The finding that multiple oral administrations of TCBZ to mice at 50 mg/kg over 5 days was safe without any demonstrable clinical signs or observable morphological effects on the main organs examined also provides the possibility of using PMB in combination with TCBZ for human use after robust testing in animal models of infection. Additionally, the fact that no resistance developed to TCBZ by *S. aureus* after 21 daily sequential in vitro sub-cultures also suggests that its synergistic antimicrobial activity with PMB against Gram-negative pathogens warrants further exploration for specific treatment of acute Gram-negative bacterial infections. This will be of further benefit in the colistin resistance era as polymyxins are among the last line of antimicrobials used to treat multidrug-resistant Gram-negative bacterial infections.

The in vivo efficacy data show that repeated treatment of mice resulted in a substantial reduction in *S. aureus* populations over the 24 h period, with regrowth occurring once treatment was withdrawn. This result suggests that TCBZ is bacteriostatic when the effective concentration in vivo is low (possibly due to poor host cell membrane penetration) and could further explain the in vitro intracellular MIC result of TCBZ against *S. aureus* ATCC 29213, where it was also found to be bacteriostatic. It is known that neutrophils are recruited to the site of invading bacterial pathogens by host pattern recognition receptors through recognition of lipoteichoic acid (LTA), a component of the cell wall of all Gram-positive bacteria [49,50]. Cell-wall-active β-lactam antimicrobials such as imipenem, flucloxacillin and cefamandole significantly enhance the release of LTA compared to protein synthesis, inhibiting drugs such as gentamicin and erythromycin [51]. Furthermore, together with glycopeptides, rifamycins, lincosamides, quinolones and fosfomycin, these β-lactam antibiotics can penetrate human neutrophils and kill and/or inhibit intracellular *S. aureus* without adversely affecting neutrophil function [51]. By contrast, we hypothesise that TCBZ does not target the bacterial cell wall and may not initiate release of LTA to the same extent. It would therefore be informative to investigate if a combination of any of these antibiotics with TCBZ could be synergistic in eradicating intracellular *S. aureus*. 

## 5. Conclusions

In this study, we showed that TCBZ, a benzimidazole anthelmintic agent, is bactericidal against coagulase-positive staphylococci, including *S. aureus* (including MRSA) and *S. pseudintermedius* (including MRSP), at relatively low concentrations and demonstrates good intracellular activity at higher concentrations. In addition, oral treatment of mice with TCBZ after systemic *S. aureus* challenge resulted in a significant reduction in *S. aureus* populations in the blood of mice up to 18 h post-infection compared to control (untreated) mice but did not clear the bacterial infection from the bloodstream, suggesting bacteriostatic activity in vivo. Future studies of pharmacokinetics and dosage optimisation of TCBZ may identify a reliable and effective oral administration regimen. 

## Figures and Tables

**Table 1:** MIC values for TCBZ derivatives against 2 MRSA and 2 VRE isolates.

Compound	Identity	MIC (µg/mL)			
		VRE 60FR	VRE 252	ATCC 49775 (MSSA)	USA300 (MRSA)
TCBZ *	Triclabendazole, 2-methyl thio	4	8	2	2
TCBZ-SO *	Triclabendazole, 2-methylsulphoxide	16	16	8	8
**TCBZ-SO_2_**	Triclabendazole, 2-methylsulphone	>256	>256	8	>256
TCBZ-SH *	Triclabendazole, 2 thio	16	8	2	4
**TCBZ-OH**	Triclabendazole, 2-hydroxy	>256	>256	>256	>256
**Ampicillin**	Ampicillin	0.125	0.5	<0.125	64

MIC test was performed in duplicate. *, Compounds selected for further screening.

**Table 2:** MIC values for TCBZ, TCBZ-SO and TCBZ-SH against 3 VRE, 3 MRSA and 2 *S. pneumoniae* isolates.

Bacterial Strain/Isolate	MIC (µg/mL) for:		
	TCBZ	TCBZ-SO	TCBZ-SH
**VRE35C**	4	32	16
**VRE60FR**	8	32	16
**VRE252**	4	16	16
**MRSA USA 300**	2	8	2
**MSSA 49775**	2	8	2
**MRSA 610**	2	16	2
***S. pneumoniae*** **A66.1**	16	>64	>64
***S. pneumoniae*** **D39**	16	>64	>64

**Table 3:** MIC values for TCBZ against 20 MRSA isolates.

Compound	Concentration (µg/mL)			
	MIC range	MIC_50_	MIC_90_	MBC
**TCBZ**	2–4	2	4	2–16
**Daptomycin**	0.25–1	0.5	0.5	ND

ND, not determined.

**Table 4:** MIC (μg/mL) values for TCBZ, PMB and in combination against Gram-negative reference strains.

Isolates	MIC (μg/mL)			Combination Effect (FICI) ^a^	DRI ^b^ PMB: TCBZ
	Single Drug		Combination		
	PMB	TCBZ	PMB: TCBZ		
***E. coli* ATCC 10763**	0.5	>256	0.125:0.25	Synergism (0.25)	4:1024
***E. coli* ATCC 25922**	0.5	>256	0.125:0.125	Synergism (0.25)	4:2048
***P. aeruginosa* PAO1**	0.5	>256	0.125:2	Synergism (0.25)	4:128
***K. pneumoniae* ATCC 33495**	0.5	>256	0.125:1	Synergism (0.25)	4:256
***K. pneumoniae* ATCC 4352**	0.5	>256	0.125:1	Synergism (0.25)	4:256
***A. baumannii* ATCC 19606**	1	>256	0.125:0.5	Synergism (0.125)	8:512
***A. baumannii* NCIMB 12457**	1	>256	0.125:1	Synergism (0.125)	8:256
***N. meningitidis* 423**	>256	32	4:16	Additivity (0.516)	64:2
***N. meningitidis* 424**	>256	32	4:16	Additivity (0.516)	64:2
***N. gonorrhoeae* ATCC 16599**	>256	>256	4:32	Additivity (0.516)	64:8
***N. gonorrhoeae* ATCC 49226**	>256	>256	4:32	Additivity (0.516)	64:8

MIC, minimum inhibitory concentration. ^a^ FICI, fractional inhibitory concentration index: synergistic, FICI ≤ 0.5; additive, 0.5 < FICI ≤ 1; indifferent, 1 < FICI ≤ 4; and antagonistic, FICI > 4. ^b^ DRI, dose-reduction index.

**Table 5:** MIC range, MIC_50_ and MIC_90_ values for TCBZ and PMB alone and in combination against 20 *E. coli*, 20 *K. pneumoniae*, 18 *A. baumannii* and 20 *P. aeruginosa* from humans.

Isolates	Values	Antimicrobial Concentration (μg/mL)				Combination Effect (FICI) ^a^	DRI ^b^	
		Single Drug		Combination				
		PMB	TCBZ	PMB	TCBZ		PMB	TCBZ
***E. coli*** **(*n* = 20)**	MIC range	0.125–1	>256	0.06–0.125	0.25–2		2–8	128–2048
	MIC_50_	0.5	>256	0.125	0.5		4	512
	MIC_90_	0.5	>256	0.125	1	Synergism (0.25)	4	256
***K. pneumoniae*** **(*n* = 20)**	MIC range	0.125–1	>256	0.06–0.5	0.5–16		2–8	16–512
	MIC_50_	0.5	>256	0.25	4		2	64
	MIC_90_	1	>256	0.5	8	Additivity (0.53)	2	32
***A. baumannii*** **(*n* = 18)**	MIC range	0.5–1	>256	0.125–0.125	0.5–2		4–8	128–512
	MIC_50_	1	>256	0.125	2		8	128
	MIC_90_	1	>256	0.125	2	Synergism (0.13)	8	128
***P. aeruginosa*** **(*n* = 20)**	MIC range	0.25–1	>256	0.06–0.25	0.125–4		2–4	64–2048
	MIC_50_	0.5	>256	0.125	2		4	128
	MIC_90_	0.5	>256	0.25	4	Additivity (0.516)	2	64

MIC, minimum inhibitory concentration. ^a^ FICI, fractional inhibitory concentration index: synergistic, FICI ≤ 0.5; additive, 0.5 < FICI ≤ 1; indifferent, 1 < FICI ≤ 4; and antagonistic, FICI > 4. ^b^ DRI, dose-reduction index.

## Data Availability

The data presented in this study are available on request from the corresponding author. The data are not publicly available due to privacy and access restrictions.

## Supplementary Materials

The following are available online at https://www.mdpi.com/article/10.3390/microorganisms9081697/s1, Figure S1. Luminescence signal comparison between groups of CD1 mice challenged IP with 1 × 10^7^ CFU of *S. aureus* (Xen29) (*n* = 6) (treated at 2, 6, 10 and 18 h post-infection). Mice were subjected to bioluminescence imaging on IVIS Lumina XRMS Series III system. Dashed horizontal line indicates limit of detection (2.0 × 10^4^ photons/s). Table S1. Gram-positive and Gram-negative bacterial collection used in this study (*n* = 138). Table S2. MIC values of TCBZ against 20 MRSA isolates. Table S3. MIC and MBC values of TCBZ against *S. pseudintermedius* isolates (*n* = 13).
