# Supplementary material for: Repurposing of the Fasciolicide Triclabendazole to Treat Infections Caused by Staphylococcus spp. and Vancomycin-Resistant Enterococci
Source: Microorganisms. 2021 Aug 10;9(8):1697. doi: 10.3390/microorganisms9081697 (PMC8398527; doi:10.3390/microorganisms9081697)
Supplement: Supplementary file 1 [file microorganisms-09-01697-s001.zip › microorganisms-1300086-supplementary.pdf]

Supplementary Materials

Repurposing of the fasciolicide triclabendazole to treat infections caused by *Staphylococcus* spp. and vancomycin-resistant enterococci

Hongfei Pi [1], Abiodun D. Ogunniyi [1], Bhumi Savaliya [1], Hang Thi Nguyen [1, 2], Stephen W. Page [3], Ernest Lacey [4], Henrietta Venter [5], Darren J. Trott [1,*]

[1] Australian Centre for Antimicrobial Resistance Ecology, School of Animal and Veterinary Sciences, Roseworthy Campus, The University of Adelaide, Roseworthy, SA 5371 Australia; hongfei.pi@adelaide.edu.au (H.P.); david.ogunniyi@adelaide.edu.au (A.D.O.); bhumi.savaliya@adelaide.edu.au (B.S.); hang.t.nguyen@adelaide.edu.au (H.T.N.);

[2] Department of Pharmacology, Toxicology, Internal Medicine and Diagnostics, Faculty of Veterinary Medicine, Vietnam National University of Agriculture, Hanoi 100000, Vietnam

[3] Luoda Pharma, Caringbah NSW 2042, Australia; swp@advet.com.au

[4] Microbial Screening Technologies Pty. Ltd, Smithfield, NSW 2164, Australia; elacey@microbialscreening.com

[5] Health and Biomedical Innovation, Clinical and Health Sciences, University of South Australia, Adelaide, SA 5000, Australia; rietie.venter@unisa.edu.au

* Correspondence: darren.trott@adelaide.edu.au

Keywords: multidrug resistance, triclabendazole, polymyxin B, bacterial pathogens, bioluminescence, sepsis

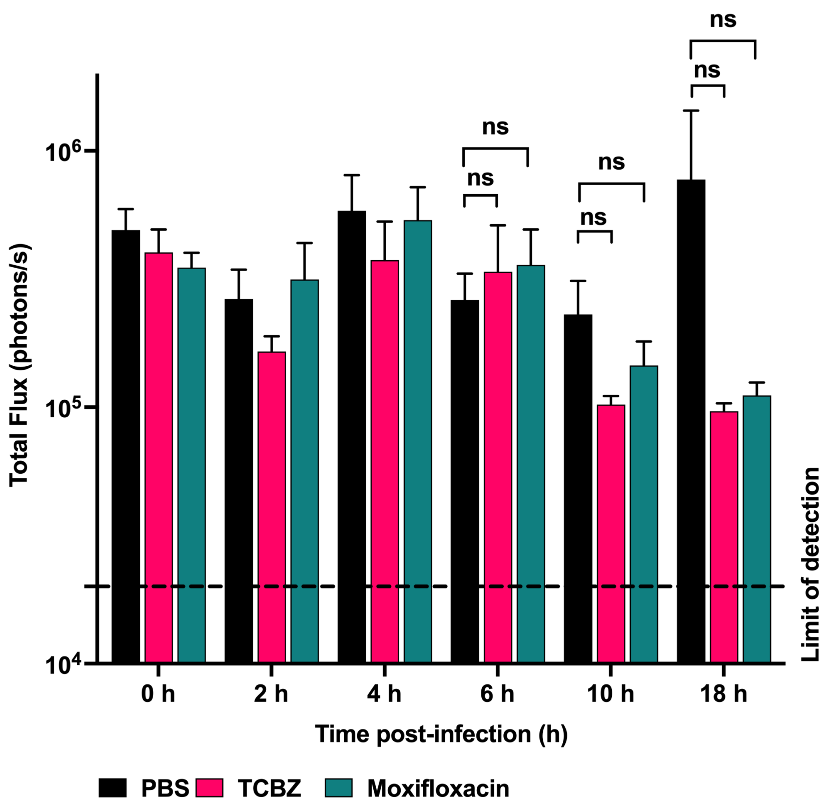

Figure S1. Luminescence signal comparison between groups of CD1 mice challenged IP with 1 × 10^7 CFU of *S. aureus* (Xen29) (n=6) (treated at 2, 6, 10 and 18 h post-infection). Mice were subjected to bioluminescence imaging on IVIS Lumina XRMS Series III system. Dashed horizontal line indicates limit of detection (2.0 × 10^4 photons/s). Data are means (±SEM) photons/s. ns = not significant (Mann-Whitney *U*-test; one-tailed).

Table S1. Gram-positive and Gram-negative bacterial collection used in this study (n=138).

**Gram positive (n = 55):**	**Gram-negative (n = 83):**
**VRE (n = 4):**	***E. coli* (n = 21):**
VRE 16C	*E. coli* ATCC 19763
VRE 35C	*E. coli* ATCC 25922
VRE 60FR	*E. coli* ATCC 10763
VRE 252	*E. coli* clinical isolates n1-n18
***S. aureus* (n = 24):**	***K. pneumoniae* (n = 20):**
S. aureus ATCC 29213	*K. pneumoniae* ATCC 33495
Bioluminescent Xen29	*K. pneumoniae* ATCC 3452
MSSA 49775	*K. pneumoniae* clinical isolates n1-n18
**MRSA (n = 21):**	***P. aeruginosa* (n = 20):**
AUS2	*P. aeruginosa* PAO1
AUS3	*P. aeruginosa* clinical isolates n1-n19
BB PVL	***A. baumannii* (n = 18):**
Classic MRSA	*A. baumannii* ATCC 19606
Irish 1	*A. baumannii* ATCC 12457
Irsih 2	*A. baumannii* clinical isolates n1-n16
NY/Japan	***N. meningitidis* (n = 2):**
QLD clone	*N. meningitidis* clinical isolate 423
ST 398	*N. meningitidis* clinical isolate 424
Taiwan	***N. gonorrhoeae* (n = 2):**
UK 15	*N. gonorrhoeae* ATCC 16599
UK 15 PVL	*N. gonorrhoeae* ATCC 49226
UK 16	
UK 17	
USA300	
WA 1	
WA 2	
WA 3	
WA 84	
WSSP	
MRSA 610	
***S. pneumoniae* (n = 2):**	
S. pneumoniae A66.1	
S. pneumoniae D39	
***S. pesudintermedius* (n = 3):**	
SP-1; SP-2; SP-3	
**MRSP (n = 10):**	
MRSP-1; MRSP-2; MRSP-3; MRSP-4; MRSP-5; MRSP-6; MRSP-7; MRSP-8; MRSP-9; MRSP-10	
**CoNS (n = 3):**	
CoNS-1; CoNS-1; CoNS-3	
***Streptococcus spp.* (n = 9):**	
***S. uberis* (n = 3):**	
SU-1; SU-2; SU-3	

***S. agalactiae* (n = 3):**	
SA-1; SA-2; SA-3	
***S. dysgalactiae* (n = 3):**	
SD-1; SD-2; SD-3	

Table S2. MIC values of TCBZ against 20 MRSA isolates.

Isolates	MIC (μg/mL) of TCBZ	MIC (μg/mL) of daptomycin
**AUS 2**	4	1
**AUS 3**	4	0.5
**BB PVL**	4	0.5
**Classic MRSA**	2	0.5
**Irish 1**	2	0.5
**Irish 2**	4	0.5
**NY/Japan**	4	0.5
**QLD clone**	4	0.25
**ST 398**	2	0.5
**Taiwan**	2	0.5
**UK 15**	2	0.5
**UK 15 PVL**	2	0.5
**UK 16**	4	0.5
**UK 17**	2	0.5
**USA 300**	2	0.5
**WA 1**	2	0.5
**WA 2**	2	0.5
**WA 3**	2	0.5
**WA 84**	2	0.5
**WSPP**	2	0.5
**MIC range**	2–4	0.25–1
**MIC_{50}**	2	0.5
**MIC_{90}**	4	0.5

Table S3. MIC and MBC values of TCBZ against *S. pseudintermedius* isolates (n = 13).

Isolates	MIC (μg/mL) of TCBZ	MBC (μg/mL) of TCBZ
**SP-1**	4	4
**SP-2**	4	4
**SP-3**	4	4
**MRSP-1**	2	4
**MRSP-2**	2	4
**MRSP-3**	4	>64
**MRSP-4**	4	16
**MRSP-5**	4	4
**MRSP-6**	4	4
**MRSP-7**	4	4
**MRSP-8**	4	4
**MRSP-9**	4	4
**MRSP-10**	4	4
**MIC range**	2-4	
**MIC_{50}**	4	
**MIC_{90}**	4	
